# Prism adaptation test before strabismus surgery in patients with decompensated esophoria and decompensated microesotropia

**DOI:** 10.1007/s10792-022-02219-3

**Published:** 2022-01-17

**Authors:** Caroline Gietzelt, Julia Fricke, Antje Neugebauer, Andrea Hedergott

**Affiliations:** Department of Ophthalmology, Faculty of Medicine and University Hospital Cologne, University of Cologne, Kerpener Str. 62, 50937 Cologne, Germany

**Keywords:** Prism adaptation test, Strabismus surgery, Decompensated esophoria, Decompensated microesotropia

## Abstract

**Purpose:**

To evaluate the effect of Prism adaptation test (PAT) on the angle of squint in decompensated esophoria (decEPH) and decompensated microesotropia (decMET).

**Methods:**

In this single-center retrospective study we reviewed the medical records of patients with the diagnosis of decEPH or decMET, aged at least 12 years, who were treated by strabismus surgery for the first time. The maximum Angle of squint (AOS) for far (F) and near (N) fixation and PAT results before surgery, as well as AOS (F) and AOS (N) after surgery and results of binocular function tests were considered. PAT included wearing a prism based on the largest angle for over 60 min.

**Results:**

100 patients (mean age 37 ± 17 years) were included in the decEPH group, 82 patients (mean age 30 ± 13 years) in the decMET group. For decEPH, before surgery AOS was 25.5 ± 8.8 pdpt (F) and 23.5 ± 9.8 pdpt (N). During PAT the AOS increased significantly by 2.7 ± 4.3 to 28.2 ± 8.6 pdpt (F) and by 4.9 ± 4.5 to 28.3 ± 9.5 pdpt (N). Altogether, in 82% of decEPH patients AOS (F) and/ or AOS (N) in- or decreased by at least 3 pdpt. For decMET, before surgery AOS was 28.6 ± 10.8 pdpt for far (F) and 30.9 ± 11.8 pdpt for near fixation (N). During PAT the AOS increased significantly by 4.2 ± 5.8 to 32.5 ± 9.5 pdpt (F) and by 3.7 ± 6.1 to 34.4 ± 9.5 pdpt (N). Altogether, in 51% of decMET patients, AOS (F) and/ or AOS (N) increased by at least 10 pdpt, therefore more than 5° which would have been maximally expected from mictrotropia, or decreased by at least 3 pdpt.

**Conclusions:**

The Prism adaptation test (PAT) showed remarkable changes in AOS in both decEPH and decMET. In patients with decEPH, the preoperative assessment of the “true AOS” under PAT reflects a pivotal requirement for successful strabismus surgery, as 82% had dose relevant angle changes ≥ 3 pdpt. For patients with decMET the preoperative prism adaptation test is especially of diagnostic value, but also 51% of decMET patients had changes in AOS beyond the expected microtropic angle (≥ 10 pdpt) or even a dose relevant angle decrease (≥ 3pdpt).

## Introduction

The Prism adaptation test (PAT) is widely used in Europe as a means to preoperatively evaluate the maximum Angle of squint (AOS) and determine the necessary dosing of strabismus surgery [1, 2]. Pichler et al. conducted a survey among Austrian strabologic surgeons and evaluated how frequently a PAT was performed prior to strabismus surgery. The results differed remarkably depending on the type of strabismus: While for intermittent exotropia, decompensated exophoria, and decompensated esophoria 90–98% of surgeons answered to perform PAT prior to strabismus surgery, in patients with infantile esotropia only 49% stated to perform PAT prior to surgery [1].

Evidence from the literature suggests that for acquired esotropia, so-called augmented surgery, in which the surgery was planned according to the prism-adapted angle of squint, leads to significantly better results than surgery which was planned without PAT [3]. However, it remained unclear, whether the increased success was really due to the prism adaptation test or simply due to an increased total amount of surgery [4] or there might have been other bias to the study [5].

One other missing factor in most studies is, that the authors do not differentiate between different types of acquired non-accommodative esotropia [6–8]. Therefore, the aim of this study was to evaluate the effect of PAT on the AOS in decompensated esophoria (decEPH) and decompensated microesotropia (decMET).

The U.S. American literature describes Acute acquired comitant esotropia (AACE) as a disease with sudden onset of large-angle esotropia with preceding intermittent diplopia [9]. Buch et al. [10] found acute accommodative strabismus (31%) and decompensated esophoria and decompensated monofixation syndrome (together 27%) to be the most common subtypes of AACE in children (1–15 years old).

Microesotropia (MET), also called fixation disparity or monofixational phoria in the U.S.A., is characterized by a small convergent angle of squint with anomalous retinal correspondence [11]. While sensory fusion may be achieved with subnormal levels of stereopsis, full motor fusion of the small manifest angle is not possible due to anomalous correspondence. Therefore, full motor fusion also cannot be achieved by prisms or surgery. Besides reduced binocular function and a small manifest esotropia revealed by cover-uncover-test, patients often show anisometropia and amblyopia. MET can decompensate into a larger angle (decompensated microesotropia (decMET)), often with diplopia, which can be corrected by prisms or surgery. Postoperatively, MET will remain [12–14].

Decompensated esophoria (decEPH) is characterized by latent esodeviations with normal binocular functions which gradually decompensate into manifest esotropia, most often associated with diplopia.

In a small sample of 26 patients, Savino et al. highlighted the importance of preoperative differentiation between decompensated microtropia (also called Monofixation syndrome (MFS)) and other forms of acquired comitant esotropia with diplopia for an accurate aesthetic and functional prognosis and appropriate surgical treatment plan [15]. Further, Ali et al. recently also drew attention to the diagnosis “decompensated esophoria” as a differential diagnosis in young adults with gradually progressive intermittent, horizontal, binocular diplopia converting to concomitant large angle esotropia [16]. Of the esophoric patients who required surgery, 71% were undercorrected after surgery. The authors did not report whether prism adaptation was performed preoperatively.

In line with a prospective AACE study of Lyons et al. also in our clinical experience, the most frequent AACE types requiring surgery in older children and young adults are decompensated microesotropia (decMET) and decompensated esophoria (decEPH) [17]. In our opinion, preoperative differentiation between the two entities is of clinical importance, as the two diagnoses have different characteristics which can influence the preoperative orthoptic examination result as well as the outcome after surgery.

The goal of this retrospective study was to evaluate the effect of PAT on the angle of squint in decMET and decEPH.

## Material and methods

### Patients

In this single-center retrospective study, we reviewed the medical records of consecutive patients treated by strabismus surgery due to decEPH or decMET between 2003 and 2019 at the center. Inclusion of patient records was stopped after a consecutive 100 cases.

Inclusion criteria for all patients were the preoperative diagnosis of decEPH or decMET, patient age of at least 12 years at the time of surgery, and the documentation of a baseline examination with documented PAT results one to three days before surgery and a follow-up examination one day after surgery. Exclusion criteria were additional neurologic symptoms and previous strabismus surgery. The eye which received the strabismus surgery was considered the study eye.

Patients diagnosed with decEPH before surgery were grouped into one subgroup while patients with the preoperative diagnosis of decMET were grouped into the other subgroup. Additional exclusion criteria for the decEPH group were additional surgery on the oblique muscles or postoperatively diagnosed microtropia.

Diagnostic criteria for decMET were an anamnestic small or even unknown convergent AOS which had converted into a larger angle with or without diplopia [11], reduced binocular functions after prism adaptation, with a persisting small manifest esotropia after prism adaptation, with a predominant leading eye.

Decompensated esophoria (decEPH) was diagnosed if latent esodeviations with normal binocular functions gradually had decompensated into manifest esotropia, usually associated with initially intermittent and gradually persisting diplopia, without amblyopia, often associated with myopia, with good stereovision after prism adaptation and without manifest deviation after prism adaptation, with normal ocular motility, regular saccades, lack of continuous incomitance, and absence of accompanying neurological symptoms (such as saccadic pursuits, dysmetric saccades, downbeat nystagmus, ataxia).

### Orthoptic and ophthalmologic examination

The baseline examination was performed one to three days before surgery. The follow-up examination was performed one day postoperatively. All examinations were conducted by experienced ophthalmologists and orthoptists. The following data have been extracted from the medical records:

- Best-corrected visual acuity (BCVA) of the study eye and fellow eye
- Refraction (in pseudophakic patients, anamnestic refraction before cataract surgery was considered) of the study eye and fellow eye
- Prism diopters of Fresnel prism or prism glasses at baseline, if worn
- Family history of strabismus
- Tests for binocular function (Bagolini striated glasses test, Lang I or II stereo test, Titmus stereo test)
- Measurement of the largest Angle of squint (AOS) by alternating prism and cover test at near (AOS(N)) and far fixation (AOS(F)) with the head in primary position before and after PAT and postoperatively

### Prism adaptation test

At baseline examination, a Prism adaptation test (PAT) was performed in every patient according to the standard operating procedure of our clinic: Based on the largest angle measured with alternating prism and cover test before PAT, single prisms were mounted on the patient’s glasses or Plano eyeglasses and worn for at least one hour. Afterward and while the patient was still wearing the eyeglasses with attached prisms, again the AOS(N) and AOS(F) were measured and the tests for binocular function were carried out (at least Bagolini Test for far and near fixation).

### Ethics and statistics

According to regional medical regulations on retrospective single-center clinical studies (§15 of the Professional code of conduct, General Medical Council for the Northern Rhine in accordance with the General Data Protection Regulation GDPR of the European Union), the Ethics Committee of the University of Cologne decided that further approval was not required for this retrospective analysis. Throughout the whole study, the declaration of Helsinki and applicable national regulations and laws were observed.

Statistical analysis was performed using Excel (Microsoft Excel for Mac, Version 15.29.1, Microsoft, USA) and SPSS (IBM SPSS Statistics, Version 25.0, IBM, USA). For metric data, mean and standard deviation were calculated. Means were compared between groups with student`s paired t-test for data with normal distribution, otherwise with Wilcoxon test. The threshold for statistical significance was set to *p* < 0.05.

## Results

We were able to identify 100 patients who met all inclusion and no exclusion criteria for the decEPH group (2008–2019) and 82 patients, who met all inclusion and no exclusion criteria for the decMET group (2003–2019). Detailed epidemiologic data concerning sex, age, family history for strabismus, eye, BCVA, refraction, and preoperative prisms (Fresnel prism or prism glasses) are summarized in Table 1.

**Table 1:** Detailed data concerning sex, age, family history for strabismus, eye, BCVA, refraction and preoperative prisms (Fresnel prism or prism glasses)

Diagnosis	„Esophoria“ dekEPH (*n* = 100)		„Mikroesotropia“ dekMET (*n* = 82)	
sex, female:male (%)	52:48		49:51	
age at day of surgery (years)	37 ± 17		30 ± 13	
mean ± SD (range)	(12–79)		(12–59)	
Family history for strabismus	27%		42%	
Study eye, right:left (%)	62:38		50:50	
	Study eyes	Fellow eyes	Study eyes	Fellow eyes
BCVA (logMAR)	0.07 ± 0.7	− 0.05 ± 0.6	0.09 ± 0.21	− 0.013 ± 0.77
mean ± SD (range)	(− 0.1 to 0.2)	(− 0.2 to 0.2)	(− 0.1 to 1.3)	(− 0.2 to 0.2)
spherical equivalent (dpt)	− 2.27 ± 2.75	− 2.12 ± 2.67	− 0.55 ± 3.39	− 0.76 ± 3.31
mean ± SD (range)	(− 11.63 to + 2.75)	(− 11.63 to + 2.5)	(− 11.88 to 7.25)	(− 11.63 to 6.75)
myopia ≥ -0.5dpt	56%		44%	
hyperopia ≥ + 1.0dpt	13%		35%	
anisometropia ≥ 1dpt	11%		16%	
prisms preoperatively (Fresnel prisms or prism glasses)	76%		23%	

In the decEPH group, there was no statistically significant difference in BCVA between the study eyes and the partner eyes (BCVA of study eyes = 0.07 ± 0.7logMar, BCVA of partner eyes = − 0.05 ± 0.6logMar; *p* = 0.064). In the decMET, group the mean BCVA of the study eyes was significantly lower than that of the partner eyes (BCVA of study eyes = 0.09 ± 0.21logMar, BCVA of partner eyes = − 0.013 ± 0.77logMar; *p* < 0.001).

76% of all decEPH patients already had Fresnel prisms or prism glasses at baseline examination (with a mean of 12 ± 10pdpt for correction of an esophoric or esotropic angle) while only 23% of all decMET patients had Fresnel prisms or prism glasses at baseline examination (with a mean of 13.5 ± 8.9pdpt).

Detailed orthoptic data and main outcome concerning AOS before and after PAT, results of the PAT, postoperative AOS, and binocular functions are summarized in Table 2.

**Table 2:** Detailed orthoptic data and main outcome concerning AOS before and after PAT, results of the PAT, postoperative AOS and binocular functions

Diagnosis	„Esophoria“ decEPH (*n* = 100)		„Mikroesotropia“ decMET (*n* = 82)	
AOS (pdpt)				
Far fixation	25.5 ± 8.8		28.6 ± 10.8	
Near fixation	23.5 ± 9.8		30.9 ± 11.8	
PAT	25.1 ± 8.6		30.17 ± 10.47	
AOS with PAT (pdpt)				
Far fixation	28.2 ± 8.6		32.5 ± 9.5	
Near fixation	28.3 ± 9.5		34.4 ± 9.5	
Increase with PAT (pdpt)				
Far fixation	2.7 ± 4.3*		4.2 ± 5.8	
Near fixation	4.9 ± 4.5*		3.7 ± 6.1	
Level of significance for Increase with PAT				
Far fixation	*p* < 0.001		*p* < 0.001	
Near fixation	*p* < 0.001		*p* < 0.001	
	Before PAT	After PAT	Before PAT	After PAT
Mean distance-near difference				
(DND = AOS(F)-AOS(N))	+ 2.0 ± 5.9	− 0.12 ± 4.0	− 2.3 ± 6.4	− 1.9 ± 3.8
Postoperative findings				
AOS (pdpt) postoperatively				
Far fixation	3.3 ± 3.5		5.3 ± 4.8	
Near fixation	2.5 ± 4.3		5.8 ± 5.7	
Level of significance for AOS reduction				
Far fixation	*p* < 0.001		*p* < 0.001	
Near fixation	*p* < 0.001		*p* < 0.001	

Binocular functions	Before surgery	After surgery	Before surgery	After surgery
Bagolini striated glasses test (%)	28	90*	13	74.4*
Lang test (%)	29		2.4	
Diplopia (%)	61	7	34	6.1
Exclusion (%)	9	3	50	19.5

*Statistically significant difference

**Fig. 1:**
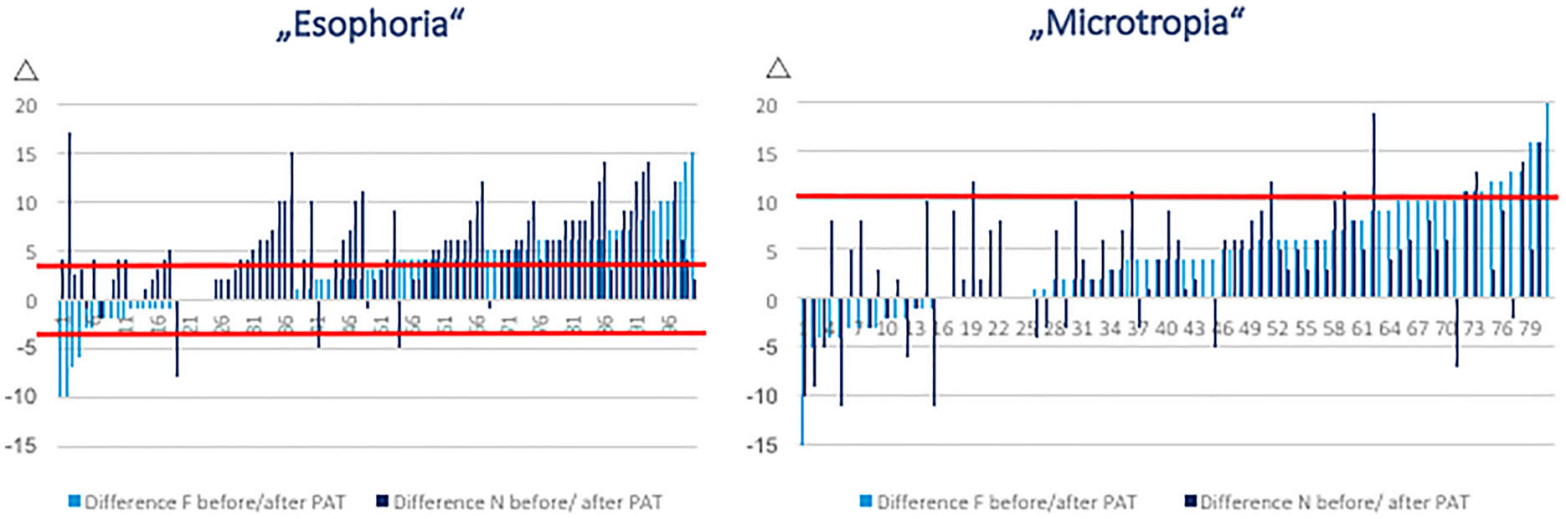
Individual change of AOS with PAT for far and near fixation for each patient

The individual changes of AOS with PAT for far and near fixation per patient and diagnosis are displayed in Fig. 1.

### Decompensated esophoria

For decEPH, before surgery AOS was 25.5 ± 8.8 pdpt (F) and 23.5 ± 9.8 pdpt (*N*). During PAT the AOS increased significantly by 2.7 ± 4.3 to 28.2 ± 8.6 pdpt (F) and by 4.9 ± 4.5 to 28.3 ± 9.5 pdpt (*N*) (*p* < 0.001 respectively).

In our clinical dosage standard, changes in AOS of more than 3 pdpt are considered relevant for dose-finding and change the amount of surgery by 1 mm recession or plication of the muscle (for patients with refraction due to axial length under 6D and correction by glasses). We analyzed whether changes with impact on dose-finding occurred after PAT in the decEPH group.

In 52% of decEPH patients, the AOS (F) enlarged at least by 3 pdpt. 27% of them had an AOS (F) increase of ≥ 3 < 6 pdpt, 18% an increase of ≥ 6 < 9 pdpt, and 7% had an increase of at least 9 pdpt. In 6%, AOS (F) was reduced by at least 3 pdpt. The AOS (N) enlarged in 69% of decEPH patients at least by 3 pdpt. 26% of them had an AOS (N) increase of ≥ 3 < 6 pdpt, 23% an increase of ≥ 6 < 9, and 20% had an increase of at least 9 pdpt. In 3%, AOS (N) was reduced by at least 3 pdpt. Altogether, in 82% of decEPH patients AOS (F) and/ or AOS (N) in- or decreased by at least 3 pdpt.

In the decEPH group, the AOS before PAT measured for far fixation was significantly higher than measured for near fixation (p = 0.001). The distance-near difference (DND) after PAT decreased significantly compared to the DND before PAT (DND (before PAT) = 2.0 ± 5.9, DND (after PAT) =  − 0.12 ± 4.0, *r* = 0.54, *p* < 0.001).

A subgroup of 76 decEPH patients wore Fresnel prisms or prism glasses preoperatively for several days to weeks (mean prism strength 15.8 ± 7.9pdpt). In most cases, the prisms corrected the AOS only partially: only 32% of showed a positive Bagolini Test (far fixation) with the prisms. The mean difference between prisms worn preoperatively and PAT was 8.9 ± 7.8 pdpt (subgroup with positive Bagolini test: 6.8 ± 5.7pdpt, subgroup with negative Bagolini test or diplopia: 10.1 ± 8.5pdpt. This difference was not statistically significant (*p* = 0.134)). The angle enlargement with PAT compared to AOS before PAT was 2.2 ± 4.4pdpt for far distance and 4.2 ± 4.4pdpt for near distance. In comparison, the 24 patients without previous Fresnel prisms or prism glasses, the PAT showed a significantly higher angle enlargement both for far and for near distance of 4.3 ± 3.6pdpt for far (*p* = 0.039), and 6.7 ± 4.2pdpt for near distance (*p* = 0.016).

### Decompensated microesotropia

For decMET, before surgery, AOS was 28.6 ± 10.8 pdpt for far (F), 30.9 ± 11.8 pdpt for near fixation (*N*). During PAT the AOS increased significantly by 4.2 ± 5.8 to 32.5 ± 9.5 pdpt (F) and by 3.7 ± 6.1 to 34.4 ± 9.5 pdpt (*N*)(*p* < 0.001 each).

In our clinical dosage standard, enlargement in the AOS of up to 10 pdpt after PAT is to be expected due to the sensory anomaly with anomalous retinal correspondence. However, any further enlargement, or overcorrection by the PAT of more than 3 pdpt, would be dose-relevant, see above. We therefore analyzed such changes in the decMET group.

In 21% of decMET patients, the AOS (F) increased at least by 10 pdpt, therefore more than the 5° which would have been expected from the mictrotropia. 11% of patients showed an angle reduction of AOS (F) of 3 pdpt and more. The AOS (N) enlarged in 15% of decMET patients by at least 10 pdpt. 16% showed an AOS (N) reduction of 3 pdpt and more. Altogether, in 51% of decMET patients, AOS (F) and/ or AOS (N) increased by at least 10 pdpt or decreased by at least 3 pdpt.

In the decMET group, the AOS measured for near fixation before PAT was significantly higher than the AOS for far fixation. There was no statistically significant difference between the DND before and after PAT (DND (before PAT) =  − 2.3 ± 6.4 pdpt, DND (after PAT) =  − 1.9 ± 3.8 pdpt, *r* = 0.39, *p* = 0,455).

### Comparison of decMET and decEPH groups

The AOS before surgery was significantly higher in the decMET group compared to the decEPH group both for far and near fixation (AOS(F) p = 0.023; AOS(N) *p* < 0.001).

Furthermore, the DND was significantly different between the decMET group and the decEPH group (*p* < 0.001): Before PAT both in the decMET group and the decEPH group there was a significant DND (*p* < 0.002 respectively). However, before PAT in the decMET group, the mean DND was -2.3 ± 6.4pdpt, meaning that overall the AOS(N) was higher than the AOS(F), the mean preoperative DND in the decEPH group was + 2.0 ± 5.9pdpt, meaning that overall the AOS(F) was higher than the AOS(N). After PAT there was still a significant DND in the decMET group (*p* < 0.001). However, in the decEPH group after PAT there was not any more a significant DND (*p* = 0.774).

In the decMET group, significantly less patients had positive postoperative Bagolini striated glasses test for far fixation than in the decEPH group (*p* = 0.001).

## Discussion

The Prism adaptation test (PAT) is used to find out the "maximum" angle of squint before eye muscle surgery that can be corrected with best binocular outcome to avoid undercorrection [1, 3]. When and how long a PAT is performed preoperatively differs from surgeon to surgeon and country to country [1]. Only recently, Zhang et al.[18] compared the therapeutic effects of surgery following prism adaptation test versus surgery alone in Acute acquired comitant esotropia (AACE). They found no significant difference in success rate between the “prism plus surgery” group and “surgery” group 12 months postoperatively. Nevertheless, surgery following prism adaptation test had better outcomes than surgery alone concerning binocular function and recurrence rate.

While many surgeons perform PAT preoperatively in Acute acquired comitant esotropia (AACE), most studies do not distinguish between different underlying causes. Therefore, our study evaluated the effect of Prism adaptation test (PAT) on the Angle of squint (AOS) in two of the main causes of AACE: decompensated esophoria (decEPH) and decompensated microesotropia (decMET).

The diagnosis of decEPH or decMET was made clinically based on multiple clinical characteristics as described above. Those characteristics could therefore also be seen in the statistical analysis of our study population. As expected, BCVA of the eye which received surgery was worse in decMET group compared to decEPH group and furthermore, the difference in BCVA between the study eye and the fellow eye was greater in the decMET group than in the decEPH group. Both binocular vision and stereo function were present in much less of the decMET patients compared to the decEPH patients. Postoperatively the Bagolini test was positive for far distance in 90% of all decEPH patients, but only 74% of all decMET patients. It should be noted that the examination was carried out on the first postoperative day.

Our study showed a significant and dose relevant change of the angle of squint with PAT for several decMET and decEPH patients:

In 52% of decEPH patients the AOS (F) enlarged at least by 3 pdpt, 7% had an AOS (F) increase of at least 9 pdpt. In only 6%, AOS (F) was reduced by at least 3 pdpt. Further, AOS (N) enlarged in 69% of decEPH patients at least by 3 pdpt. Altogether, AOS (F) and/ or AOS (N) in- or decreased by at least 3 pdpt in 82% of decEPH patients. Angle changes of 3 prisms require a dose adjustment of 1 mm for eye muscle surgery and are of clinical relevance. Therefore, in patients with decEPH, the PAT is essential for evaluation of the largest angle of squint and correct dose-finding for surgery to avoid undercorrections. This is in line with the existing literature which showed an increase of AOS under PAT in 56–81% of all patients with acquired esotropia [2, 19].

In 21% of the decMET patients, AOS (F) increased by at least 10 pdpt, therefore more than the 5° which would have been expected from the mictrotropia. 11% showed an angle reduction of 3 pdpt and more. The AOS (N) enlarged by at least 10 pdpt in 15% of decMET patients. 16% showed an AOS (N) reduction of at least 3 pdpt. Altogether, AOS (F) and/ or AOS (N) increased by at least 10 pdpt or decreased by at least 3 pdpt in 51% of decMET patients. Therefore, the change under PAT is clinically relevant not only for patients with decEPH, but also for almost half of the patients with decMET with need for adjusted dosing of the surgery. In patients with decMET the PAT is also a diagnostic tool: a positive Bagolini test with a persisting small manifest esotropia under PAT strongly supports the diagnosis of microtropia.

Before PAT there was a significant Distance-near difference (DND) in both groups (*p* < 0.002 respectively). However, it differed significantly between both groups: while in the decMET patients the AOS(F) was lower than (N), it was the other way round in decEPH patients. The DND in decMET patients could not be influenced by the PAT. However, in the decEPH group, the PAT lowered the DND significantly, resulting in a no longer significant DND after PAT in the decEPH group.

In the existing literature, there is only limited information on PAT in patients with DND with larger AOS(N) than AOS(F). In one study on PAT in young patients (aged 2–18 years) with esotropia with a DND with larger AOS(N) than AOS(F), Kutschke et al. found that augmented surgery on the basis of the enlarged angle under PAT did not lead to a better surgical success rate compared to surgery on the basis of the original near angle [20]. In our decMET group, there also was a DND with AOS(N) > AOS(F). However, Kutschke et al. included patients with the preoperative diagnosis of partially accommodative esotropia, acquired esotropia (without further differentiation), and congenital esotropia, so the results cannot be compared fully. Savino et al. compared the sensory status in patients with AACE type I/ II [21] to patients with decompensated monofixation syndrome [15]. Type I AACE (Swan type) is considered due to the interruption of fusion caused by anti-amblyopic occlusion therapy in children without a significant hyperopic refractive error. Type II AACE (Burian–Franceschetti type) is characterized by acute onset of concomitant strabismus, often associated with diplopia without an accommodative component even in cases of hyperopia and in the absence of neurological disorders; this esotropia seems to be associated with physical and psychic shock. The patients with monofixation syndrome like our patients showed a larger AOS(N) than AOS(F), angle enlargement under PAT, and after further prism increase, almost all patients still had a residual detectable angle of deviation. The abnormal retinal correspondence in patients with decMET causes the angle of squint to further increase as higher and higher prisms are applied during alternating prism and cover test.

In the previous literature there is a very inhomogeneous approach to PAT with time spans for PAT reaching from one hour [22] to 3.5 weeks [3] or even longer [1]. Building up of prisms over weeks before surgery means more visits in the clinic and can often be stressful for patients, especially when they are of young age. Therefore, it seems desirable to shorten the duration of PAT. Altmann et al. examined patients with acquired esotropia under PAT. The angle was built up until it was stable and controlled after 24 h, 4 days, and 7 days. They reported a stable angle in 94% of the patients in 4- and 7- day visits compared to the 24-h visit. Ela-Dalmann et al. found a similar outcome in patients with acquired esotropia after motor-fusion-test and 1-h prism adaptation compared to the results of the Prism Adaptation Study with several weekly visits to adopt PAT.

While most authors increased the PAT multiple times over a certain time span [23], in our study, only one prism strength was applied for PAT for one hour, and no further increase was performed. However, 76% of all decEPH patients already wore Fresnel prisms or prism glasses at the baseline examination, respectively 22% of all decMET patients–not for prism increase though, but for correction of diplopia. Our study showed that the decEPH patients without previous Fresnel prisms or prism glasses showed a significantly higher angle enlargement both for far and for near distance under PAT.

A limitation of our study is that misdiagnosis of decEPH and decMET cannot be avoided completely. Typical difficulties in the diagnosis include young age of patients with unreliable results of orthoptic and stereo function tests [24]. Therefore, to keep the rate of misdiagnosis as low as possible, we excluded patients younger than 12 years of age as well as patients with unreliable orthoptic exam.

Beyond the useful information yielded by PAT and focused on in this study, a prognosis of postoperative binocular functions or the risk of diplopia can be made using PAT.

## Conclusions

The prism adaptation test showed remarkable changes in the orthoptic parameters of patients with decEPH and decMET.

In summary, we conclude that in patients with decEPH the preoperative assessment of the “true AOS” under PAT reflects a pivotal requirement for successful strabismus surgery, as 82% had an angle in- or decrease ≥ 3 pdpt. For patients with decMET the preoperative prism adaptation test is especially of diagnostic value, but also 51% of decMET patients had changes in AOS beyond the expected microtropic angle (≥ 10 pdpt) or even a dose relevant angle decrease (≥ 3pdpt).

## Data Availability

The authors have full control of all primary data and they agree to allow International Ophthalmology to review their data upon request.

## Code availability

via corresponding author.
